# Second ISCB Latin American Student Council Symposium (LA-SCS) 2016

**DOI:** 10.12688/f1000research.12321.1

**Published:** 2017-08-16

**Authors:** Alexander Miguel Monzon, Marcia A. Hasenahuer, Estefanía Mancini, Nilson Coimbra, Fiorella Cravero, Javier Cáceres-Molina, César A. Ramírez-Sarmiento, Nicolas Palopoli, Pieter Meysman, R. Gonzalo Parra

**Affiliations:** 1 Structural Bioinformatics Group, Departamento de Ciencia y Tecnología, Universidad Nacional de Quilmes, Buenos Aires, B1876BXD, Argentina; 2 Regulation of Alternative pre-mRNA Splicing during Cell Differentiation, Development and Disease, Centre for Genomic Regulation, Barcelona, Spain; 3 Department of Bioinformatics, Institute of Biological Sciences, Universidade Federal de Minas Gerais (UFMG), Belo Horizonte, Minas Gerais, 31270-90, Brazil; 4 Chemoinformatics Group, Process Engineering PLAPIQUI (UNS-CONICET), Bahía Blanca, 8000, Argentina; 5 Laboratorio de Fisiología y Genómica de Frutales, Centro de Biotecnología Vegetal (CBV), Universidad Andrés Bello, Santiago, Chile; 6 Institute for Biological and Medical Engineering, Schools of Engineering, Medicine and Biological Sciences, Pontificia Universidad Católica de Chile, Santiago, Chile; 7 Structural Bioinformatics Unit, Fundación Instituto Leloir, IIBBA-CONICET, Buenos Aires, C1405BWE, Argentina; 8 Biomedical Informatics Research Center Antwerp (biomina), University of Antwerp, Antwerp, 2000, Belgium; 9 Advanced Database Research and Modelling (ADReM), Department of Mathematics and Computer Science, University of Antwerp, Antwerp, 2000, Belgium; 10 Quantitative and Computational Biology Group, Max Planck Institute for Biophysical Chemistry, Göttingen, 37077, Germany

**Keywords:** bioinformatics, education, ISCB, Student Council, symposium

## Abstract

This report summarizes the scientific content and activities of the second edition of the Latin American Symposium (LA-SCS), organized by the Student Council (SC) of the International Society for Computational Biology (ISCB), held in conjunction with the Fourth Latin American conference from the International Society for Computational Biology (ISCB-LA 2016) in Buenos Aires, Argentina, on November 19, 2016.

## Introduction

The Student Council (SC), part of the International Society for Computational Biology (ISCB), is a global organization that aims to nurture and impulse the next generation of bioinformaticians and computational biologists. The SC is composed of young scientists at all levels, from undergraduate, masters and PhD students to postdocs; coming from all disciplines in the field. Among the different activities that are managed by the SC, the most important ones consist of a set of symposia that are organized as satellite events of the different ISCB conferences. For more than a decade, the Student Council Symposium (SCS) has been annually organized ^1– 9^. More recently, as the organization became bigger and different Regional Student Groups (RSGs) were created in more and more countries, continental versions for the SCS started to be organized. Since 2010, the European Student Council Symposium (ESCS) is bi-annually organized as a satellite event of the European Conference of Computational Biology (ECCB) ^4, 6, 8^. In parallel, the Latin American SC community has steadily grown, with the creation of RSG Argentina in 2012. In 2014, the first edition for the Latin American Student Council Symposium (LA-SCS) was held in Belo Horizonte, Brasil ^10^. Since then, a total of five RSGs have been created in the region: RSG Argentina, RSG Brasil, RSG Chile, RSG Colombia and RSG Mexico. Two years later, in 2016, a team headed by Alexander Monzon from RSG Argentina as chair and Javier Caceres from RSG Chile as vice chair, organized the second LA-SCS. This edition was held in Buenos Aires, Argentina on November 19, 2016 as a satellite event to the Fourth Latin American conference from the International Society for Computational Biology (ISCB-LA 2016) ^11^.

## Second Latin America Student Council Symposium in Buenos Aires, Argentina

**Figure 1.**
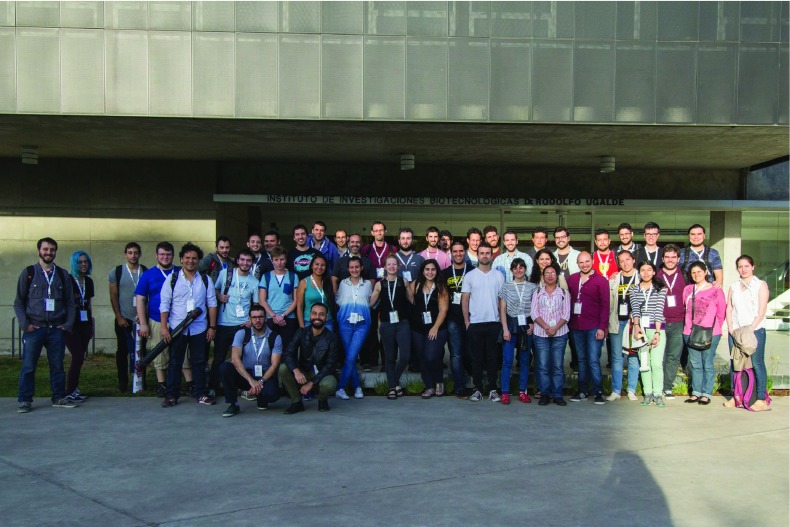
Delegates who attended to the second LA-SCS 2016.

The format of the Latin American Student Council Symposium is a day-long meeting preceding the main ISCB-LA conference. The main goals of this meeting include creating opportunities for young researchers to meet peers from all over the world, promoting the exchange of ideas and providing networking opportunities. In total, there were 65 attendees from different countries in the region and other continents as well ( Figure 1). Travel fellowships by uBiome allowed promising researchers to attend different events during the week.

The second LA-SCS had the pleasure to welcome two renowned scientists as keynote speakers: Dr. Seán O’Donoghue from the Australia's Commonwealth Scientific and Industrial Research Organisation (CSIRO) and Prof. Ruth Nussinov from the National Cancer Institute, Center for Cancer Research (CCR), United States of America and Tel Aviv University, Israel.

The symposium received 40 submissions from students that were peer-reviewed by 14 independent reviewers. 13 abstracts were selected for oral presentation and 27 additional abstracts were accepted for poster presentations.

Abstracts of poster presentations are available online in the symposium booklet ( http://lascs2016.iscbsc.org/lascs2016-booklet).

## Keynote speakers

The first keynote speaker was Dr. Seán O’Donoghue who is a senior principal research scientist in Australia's Commonwealth Scientific and Industrial Research Organisation (CSIRO) as well as group leader at the Garvan Institute of Medical Research in Sydney, Australia. His talk was entitled “Bioinformatics: a happy hunting ground for data scientists” in which he elaborated upon interesting highlights and turning points in his career which brought him to become a bioinformatician. The aim of his talk was to give students a good piece of advice, specifically focused on how the decisions made during the career help to find one’s own way in bioinformatics.

The second keynote presentation was delivered by Prof. Ruth Nussinov who is senior investigator in the Cancer and Inflammation Program, CCR, USA and a professor emeritus in the Department of Human Genetics, School of Medicine, Tel Aviv University, Tel Aviv, Israel. She also serves as Editor-in-Chief of the journal PLoS Computational Biology. She is a world reference in structural biology and bioinformatics and her talk was centered on modeling protein-protein interactions for peptide targeting. She went into detail about PRISM ^12^, an application that uses a novel prediction algorithm for protein-protein interactions.

## Student and early-career researchers’ presentations

The student presentations covered a wide range of topics in computational biology. The first student talk was presented by Yesid Cuesta, who shared an integrative method to unravel host-parasite interactomes based on an orthology approach, to understand parasite infection and local adaptation within the host. This could help to identify drug targets among genome sequence and provide a better understanding of parasite evolution behind infectious diseases.

Ariel Aptekmann reported a positive correlation between core promoter information content and optimal growth temperature in archaeal organisms, suggesting selective pressures towards binding sites with higher binding affinity to the proteins. Also, Aptekmann suggested to extend the molecular information theory between the Rsequence and Rfrequency measures in order to take into account the effect of temperature.

Emilio Fenoy presented the NetPhosPan software, a phosphorylation site predictor. This tool is based on feed-forward and a long-short term memory neural network, which extracts information from both ligand and receptor sequences. It has a higher accuracy in small datasets and uncharacterized kinases, compared to other methods.

Osvaldo Burastero described how the autophosphorylation mechanism in histidine kinases could be studied using QM/MM hybrid technics, by the analysis of different quantum level approximations and evaluation of several possible reaction mechanisms to find a concerted one-step mechanism.

Finishing the morning session, Tadeo Enrique Saldaño explored the vibrations of Human transthyretin. He found that the thyroxine hormone generates a significant change in the vibration level of the tetramer that could be partly responsible for its stabilization.

After the lunch break Daniel Almonacid and Juan Pablo Cárdenas from uBiome, the second LA-SCS platinum sponsor, gave a tech talk sharing their experiences of working in a microbial genomics company and their research career leading up to it. UBiome is a pioneer company in the newborn era of the microbiome-based precision medicine. During the tech talk, delegates were invited to collect samples of their own saliva using provisional kits. Results of their microbiome analyses were made accessible through the QR code on the uBiome website.

After the tech talk, Maria Freiberger explored if enzyme's catalytic sites are enriched in energetically conflictive (frustrated) interactions. By applying the Frustratometer software on all Catalytic Site Atlas structures, she found that residues that surround the catalytic sites or are in close proximity to metal cofactor binding sites were enriched in highly frustrated interactions. This was shown in a well-controlled study of the beta-lactamase family, observing that residues at the catalytic site were systematically in an energetic conflict with their environment.

Diego Zea shared his study which showed how large the structural space, implicitly encoded in a multiple sequence alignment of a protein family is. This large structural space leaves evolutionary signals which can be misinterpreted when only one structure from the family is analysed. Using at least one structure from four different sequence clusters, at 62% identity, it is possible to get a better description of the structural space of the family that can help in the understanding of the evolutionary signals.

Franco Simonetti presented how protein families within superfamilies’ can be clustered by coevolution residue networks. These findings provide a base to develop novel computational methods using these residues to better classify protein families with respect to their functionality.

Elin Teppa reported that conservation and coevolution at the protein-protein interface increase by the number of interacting partners, suggesting that constraints in a given position and changes in the sequence are directly related, providing novel information at the protein interface and protein-protein interaction.

Pieter Meysman presented a computational interaction model to study the affinity of the varicella-zoster virus (VZV) peptides under different HLA variants. His results strongly support the hypothesis that one of the possible underlying causes of the VZV disease severity and susceptibility is a suboptimal anti-VZV immune response due to weak HLA-binding peptide affinity.

Juan Pablo Bustamante showed the VarQ tool for structural analysis of protein variation. The software was built considering both available structural analysis tools and the Ruffus workflow system. The goal of VarQ is to help understand, analyze and discriminate the possible effect of annotated protein variations.

In the final oral presentation of the day, Soledad Ochoa reported a better cancer classification method based on mutational patterns of loci in different cancer types. This classification framework not only improves diagnosis but also has the potential for making treatment recommendations, based on similar cancer types.

## Satellite workshop

Following the format of the first LA-SCS 2014, a satellite workshop was organised to accompany LA-SCS.

We consider these kind of activities an excellent opportunity to promote knowledge exchange between students coming from different areas, such as computer science and biological careers. Taking advantage of this heterogeneity, we give PhD. students, postdocs and researchers the chance to offer workshops arranged as basic one-day courses for students, before or after the symposium day.

This time, Dr. Seán O’Donoghue and Dr. Alan Bush (from Universidad Nacional de Buenos Aires) organized and presented an eight-hour workshop entitled: “DataViz Workshop: Data Visualisation Methods and Tools - A Practical Guide”, which was held on November 18th. A total of 35 students attended the workshop. The course was structured in two blocks. First, Dr. Seán O’Donoghue offered a tour about the state-of-the-art methods and practices for turning data into insightful visualisations in order to tell compelling stories, using principles of human visual perception with modern methods and tools. He presented different tools for visualizing hierarchical, categorical, time-serial and multidimensional data, and strategies for tackling the problem of large and complex data. Then, Dr. Alan Bush presented an introduction to ggplot2, a powerful library programmed in the R programming language for generating graphics and data visualization. In a “hands-on” modality, the course went through the analysis of some examples, tutorials and practical exercises.

## Award winners

Winners for the Best Poster/Presentation Awards sponsored by F1000Research were selected by non-compulsory plurality vote of the attendees who filled up a blank ballot with the names or poster numbers of their candidates. The Outstanding Presentation Prizes were received by Emilio Fenoy (Universidad Nacional de San Martín - CONICET, Argentina), who presented “NetPhosPan: a pan-specific predictor for phosphorylation site predictions”; Lionel Uran Landaburu (Universidad Nacional de San Martín - CONICET, Argentina) for his work “Updates to the TDR Targets chemogenomics database”; and Elin Teppa (Fundación Instituto Leloir - CONICET, Argentina) who was selected for his oral talk on “Conservation and coevolution at the protein-protein interface increase with the number of interacting partners”. All winners received a certificate and discounts on article publications costs in the F1000Research open access platform.

## Conclusions

In 2014, the LA-SCS was organized for the first time as an attempt to gather together the students, postdocs and young researchers in the field of Bioinformatics and Computational Biology from Latin America. Back then, organization was led by a few representatives from RSG Argentina, the only active one in the region, and supervised by external members from the SC executive team. Many doubts appeared along the process and several obstacles arose due to the lack of local collaborators in the organizing country and the surrounding ones. Thankfully, due to great effort and support from different newly discovered volunteers, everything went smoothly. Beyond the success for that event, the most important thing was to pave the road towards setting an international network of motivated students that aimed to increase the presence of the SC in the Latin American continent. Two years later, with five RSGs in Argentina, Brazil, Chile, Mexico and Colombia, and an extended network of collaborations through virtual channels, the horizons for this new edition were quite promising. As we envisioned in the highlights from the first LA-SCS ^10^, many challenges appeared but thanks to the previous experience and the support of excellent regional collaborators, we were able to overcome most of them and minimize the impact of those that were out of our control. We have learned invaluable lessons from the successful decisions but maybe even more, from our mistakes ^13^. The experience gained will help us to continue growing as a continental collective towards bigger projects in the near future.

The third LA-SCS will be organized in 2018 in Colombia, and although the bar is now set high, we are confident that a new team will take over and repeat the success from this edition. We encourage all students from the Latin American continent to get in touch with the ISCB Student Council representatives in their countries or close ones in order to participate in the third LA-SCS organization and become a part of the development of our regional Bioinformatics and Computational Biology community.
